# Decision-making of artificial nutrition and hydration in hospice care: A qualitative study of Chinese patients and family caregivers

**DOI:** 10.1016/j.apjon.2025.100744

**Published:** 2025-06-18

**Authors:** Yunrong Li, Yuan Ji, Tiantian Wang, Bo Yang, Fulin Gao, Liuliu Zhang, Guoren Zhou, Yun Zhao

**Affiliations:** a Nursing Department, The Affiliated Cancer Hospital of Nanjing Medical University & Jiangsu Cancer Hospital & Jiangsu Institute of Cancer Research, Nanjing, China; b Department of General Surgery, The Affiliated Cancer Hospital of Nanjing Medical University & Jiangsu Cancer Hospital & Jiangsu Institute of Cancer Research, Nanjing, China; c Department of Thoracic Surgery, The Affiliated Cancer Hospital of Nanjing Medical University & Jiangsu Cancer Hospital & Jiangsu Institute of Cancer Research, Nanjing, China; d Department of Oncology, The Affiliated Cancer Hospital of Nanjing Medical University & Jiangsu Cancer Hospital & Jiangsu Institute of Cancer Research, Nanjing, China

**Keywords:** Hospice care, Parenteral nutrition, Enteral nutrition, Decision-making, Grounded theory, Qualitative research

## Abstract

**Objective:**

Decision-making of artificial nutrition and hydration (ANH) is a complex ethical and emotional decision in hospice care, significantly impacting patients' comfort and quality of life at the end of life. This study explored behaviors about decision-making of ANH in hospice patients and their family caregivers, aiming to identify the trajectory and influencing factors of the decision.

**Methods:**

This study employed constructivist grounded theory. Using purposive and theoretical sampling methods, in-depth interviews were conducted with 21 hospice patients and family caregivers in three Grade IIIA hospitals in Mainland China. Thematic analysis involved initial coding, focused coding, and theoretical coding to develop main categories and core categories.

**Results:**

This study identified a core category, alongside two main categories. A framework titled “The decision-making process of ANH for hospice patients and family caregivers” was developed. This decision-making began with the detection of malnutrition and progressed through five core stages, including “symptom shock”, “risk trade-offs”, “goal formation”, “final decision”, and “moral distress”. Multiple individual, medical, and social factors influenced the final decision-making process of ANH.

**Conclusions:**

Through the framework, this study can enhance health care providers' understanding of the decision-making process and also can aid in tailoring support to align treatment choices with patients' preferences. The study highlights the interplay between emotional and rational aspects in decision-making of ANH and emphasizes the need for health care providers to recognize individual, medical, and social factors. These findings can enhance the decision-making experience of hospice patients and family caregivers, ultimately improving the quality of hospice care.

## Introduction

With the aging population and rising prevalence of chronic illnesses, the demand for hospice care is rapidly increasing. According to the World Health Organization,^1^ approximately 56.8 million people worldwide require hospice care annually. Anorexia and dysphagia are common symptoms in hospice care for terminal patients, especially those with some gastrointestinal malignancies.^2^^,^^3^ This often forces patients and their family caregivers to make critical decisions about whether to initiate artificial nutrition and hydration (ANH). ANH is a medical intervention that provides nutritional support through parenteral (intravenous) or enteral (nasogastric or gastrostomy tubes) routes.^4^

The previous study suggested that ANH reduces physical discomfort due to nutritional and hydration deficiencies and reduces the risk of dehydration-related complications, potentially preventing premature death in certain patients.^5^ Additionally, maintaining feeding-related rituals through ANH has been shown to help family caregivers cope by preserving a sense of normalcy and reducing psychological stress during difficult times.^6^ However, research also indicated that patients in the terminal phase may no longer require or be able to process ANH, leading to an increased risk of complications compared to those receiving ANH for non-terminal conditions.^7^ In current practice, the choice between “prolonging life” and “quality of life” can create uncertainty and stress for family caregivers, this may lead to decision-making delay or decision-making avoidance, ultimately affecting patients' outcomes.^8^^,^^9^ As an alternative medical intervention, the use of ANH in hospice care is a complex and multi-dimensional issue.

Decision support can enhance patients' and their families' understanding of ANH, reduce the burden of decision-making, and help avoid unnecessary medical interventions.^10^ Although the European Society for Clinical Nutrition and Metabolism and the American Society for Parenteral and Enteral Nutrition have issued position papers on ANH,^11^^,^^12^ providing recommendations for preventing and resolving dilemmas in decision-making, the actual decision-making process remains fraught with challenges. Firstly, laws and regulations differ across countries and regions regarding the definition and management of ANH. For example, South Korea’s Life-Sustaining Treatment Decisions Act stipulates that ANH cannot be withheld or withdrawn, as it is considered part of primary care.^13^ In contrast, Taiwan’s Patient Right to Autonomy Act permits the withholding or withdrawal of ANH in conditions such as irreversible coma, vegetative state, severe dementia, or unbearable pain.^14^ Secondly, limited participation of hospice patients and their families in end-of-life decision-making is a pressing issue. While many countries are transitioning toward a patient-centered shared decision-making model to ensure hospice patients' rights to informed consent and self-determination,^15^ studies show persistent gaps in practice. Research in the UK found that health care professionals (HCPs) did not always included patients in the decision-making of ANH.^16^ Similarly, a study in Israel also revealed that most family caregivers stated that they had never discussed ANH with HCPs.^17^

In Chinese culture, ANH is not merely a clinical intervention but also carries profound symbolic meaning. Providing nutrition and hydration is often perceived as a fundamental expression of care.^18^ Moreover, influenced by the Confucian virtue of filial piety, sons and daughters often feel a moral obligation to care for their elderly or ill parents, especially in hospice care.^19^ As a result, maintaining ANH may be viewed as fulfilling this filial duty. Furthermore, the collectivist orientation of Chinese culture places strong emphasis on family involvement. Family caregivers are often highly engaged—or even take the lead–in decision-making to demonstrate their commitment to family responsibilities.^20^ Therefore, examining the decision-making of ANH within the framework of Chinese culture is of particular significance, as it can inform the development of culturally sensitive nursing strategies.

In recent years, decision-making in hospice care has received growing scholarly attention. However, most existing studies focus on Western countries or the Taiwan region, with relatively limited research on Mainland China. Furthermore, many studies have overlooked the critical role of family members in the decision-making process, limiting their relevance to clinical practice. Most existing researches have also relied on cross-sectional designs, failing to capture the evolving nature of decision-making and providing an incomplete understanding of influencing factors. To address these gaps, the present study aimed to conduct a qualitative investigation in hospice wards in Mainland China to explore the decision-making process of ANH from the dual perspectives of patients and family caregivers. Considering the prevalence and high incidence of early malnutrition among patients with gastrointestinal cancer, this study takes such patients and their family caregivers as the main research subjects. Particular attention was given to the influence of Chinese cultural factors such as family values and filial piety. The goal was to construct a complete and contextually appropriate framework for decision-making of ANH that reflects local realities. This framework not only provides a theoretical foundation for the development of decision-making support in Mainland China but also enriches cross-cultural discourse on decision-making of ANH, ultimately contributing to more adaptive and culturally responsive hospice care practices.

## Methods

### Research aim

This study aimed to develop a conceptual framework for understanding the coping trajectory of hospice patients and their family caregivers during the decision-making process of ANH, as well as the factors influencing their decisions.

### Design

Decision-making of ANH in hospice care involves stakeholders, including patients, family caregivers, and HCPs. However, there is currently no established theoretical framework for understanding the decision-making process of ANH in China. To address this gap, this study adopted Charmaz’s constructivist grounded theory^21^ as both its research framework and analytical approach, based on the philosophical foundation of symbolic interactionism. Charmaz’s constructivist grounded theory claims that theories emerge through interactions between researchers and participants, emphasizing the role of social context, interpersonal dynamics, and participants' perspectives.^22^ This approach was particularly relevant for analyzing the interactions between patients, family caregivers, and HCPs in hospice care within the cultural and health care landscape of China. By applying this methodology, the study aimed to systematically reveal the psychological conflicts, values and decision-making behaviors of ANH, thereby generating a theoretical framework to guide clinical practice.

### Participants

Study details were conveyed to potential participants by doctors and nurses in the hospice ward. These HCPs provided both verbal information and written notices during daily rounds and nursing care. Patients or their family caregivers who expressed interest could contact the HCPs directly. At the start of the study, purposive sampling was used. The researcher approached individuals from the target population, explained the purpose of the study, and obtained informed consent. Following consent, participants completed a general information questionnaire that collected data on age, gender, relationship to the patient, education level, and degree of malnutrition. Subsequently, interview times and locations were scheduled. Participant inclusion and exclusion criteria are provided in Table 1. In line with the principle of maximum variation sampling, the research team aimed to ensure diversity in literacy levels, disease types, and nutritional status among initial participants. For instance, the first three interviewees represented a range of educational backgrounds (from junior high school to undergraduate level), included patients with digestive and non-digestive system diseases, and had varying degrees of malnutrition. As data collection progressed, theoretical sampling was adopted. This allowed the research team to refine participant selection based on emerging categories and to validate relationships among these categories. For example, early interviews revealed that “the number of children the patient had” was a significant theme. In response, the team selected a decision-maker who was an only child for an in-depth interview. Theoretical sampling was also used to explore other emerging themes, such as the potential influence of age or economic status on decision-making outcomes. The final sample size was determined based on the principle of data saturation.^23^ Saturation refers to the point at which no new or relevant data emerge concerning a category, the categories are well developed in terms of their properties and dimensions, and the relationships between categories are clearly established.^24^ A total of 21 participants (8 hospice patients and 13 family caregivers) were ultimately included in the study and were labeled M1-M21. The general information of patients (*n* ​= ​8) and family caregivers (*n* ​= ​13) are detailed in Table 2.

**Table 1:** Inclusion and exclusion criteria for study participants.

Participants	Inclusion criteria	Exclusion criteria
**Patients**	1. Estimated prognosis ⩽ 6 months (assessment by clinical team) and refused curative treatment	1. Unaware of disease diagnosis
	2. Being older than 18 years old	2. Refused to participate in this study
	3. Conscious and without mental disorder	3. Failed to complete the interview
	4. Providing informed consent and voluntary participation	
**Family caregivers**	1. Family member or friend for hospice patients who identifies as a caregiver (current or former)	Failed to complete the interview
	2. Being older than 18 years old	
	3. Conscious and without mental disorder	
	4. Providing informed consent and voluntary participation

**Table 2:** Demographic profile of patients and family caregivers.

Characteristic	Patients (*n* ​= ​8)	Family caregivers (*n* ​= ​13)
**Sex**		
Male	4 (50.0%)	3 (23.1%)
Female	4 (50.0%)	10 (76.9%)
**Age (years)**		
18–30	–	–
31–50	1 (12.5%)	4 (30.8%)
51–65	3 (37.5%)	6 (46.2%)
≥ 66	4 (50.0%)	3 (23.1%)
**Relationship to patient**		
Spouse		3 (23.1%)
Daughter		6 (46.2%)
Son		2 (15.4%)
Sibling		1 (7.7%)
Parent		1 (7.7%)
Other		–
**Household per capita monthly income**		
< ¥3001	3 (37.5%)	2 (15.4%)
¥3001–6000	2 (25.0%)	3 (23.1%)
¥6001–9000	2 (25.0%)	4 (30.8%)
> ¥9000	1 (12.5%)	4 (30.8%)
**Number of offspring**		
0	–	–
1	6 (75.0%)	4 (30.8%)
≥ 2	2 (25.0%)	9 (69.2%)
**Occupation**		
Freelance	1 (12.5%)	2 (15.4%)
Employee	4 (50.0%)	7 (53.8%)
Farmer	1 (12.5%)	1 (7.7%)
None	2 (25.0%)	3 (23.1%)
**Education**		
Junior high school and lower	4 (50.0%)	6 (46.2%)
Senior high school	2 (25.0%)	1 (7.7%)
Junior college	1 (12.5%)	1 (7.7%)
Undergraduate and higher	1 (12.5%)	5 (38.5%)
**Religion**		
Yes	–	1 (7.7%)
No	8 (100.0%)	12 (92.3%)
**Diagnosis of the patient**^a^		
Gastrointestinal cancer	8 (100.0%)	7 (53.8%)
Lung cancer	–	1 (7.7%)
Renal cancer	–	1 (7.7%)
Prostate cancer	–	1 (7.7%)
COPD	–	2 (15.4%)
AD	–	1 (7.7%)
**Medical insurance type of the patient**^b^		
The new rural cooperative medical insurance	2 (25.0%)	1 (7.7%)
Self-financing	1 (12.5%)	1 (7.7%)
Insurance for urban residents	5 (62.5%)	11 (84.6%)
**Current feeding pattern of the patient**^c^		
Tube feeding	–	5 (38.5%)
Oral feeding	3 (37.5%)	–
PN	–	3 (23.1%)
Oral feeding ​+ ​PN	5 (62.5%)	4 (30.8%)
Tube feeding ​+ ​PN	–	1 (7.7%)

COPD, chronic obstructive pulmonary disease; AD, Alzheimer’s disease; PN, parenteral nutrition.

a For the “Family caregivers” column, data refer to the patients under the family caregivers' care.

b For the “Family caregivers” column, data refer to the patients under the family caregivers' care.

c For the “Family caregivers” column, data refer to the patients under the family caregivers' care.

### Data collection

The interview guide was developed through a review of relevant literature and consultations with experts in nutrition and hospice care. Following the completion of a preliminary version, pilot interviews were conducted with one hospice patient and one family caregiver to assess the guide’s effectiveness and feasibility. Throughout the interview process, the guide was continuously refined based on emerging themes. For example, the emotional burden associated with decision-making was identified as an important theme during early interviews. As a result, additional questions addressing emotional support and psychological distress were incorporated into subsequent interviews. This dynamic approach ensured comprehensive data collection. The final interview guide is presented in Table 3. Between September 2024 and December 2024, the first author (a female master’s student) volunteered in hospice wards at three Grade IIIA hospitals in Nanjing, Jiangsu Province, China. During this period, she accompanied doctors on rounds, engaged in patient and family caregiver support, and observed their daily interactions. Field notes and diaries were maintained to document these observations. Interviews were conducted face-to-face in a quiet, private nurse-patient communication room. The interviews were semi-structured, allowing for flexible responses in questioning and follow-up on key topics. Non-verbal behaviors were also observed and recorded. Each interview lasted between 15 and 60 min. Interviews were transcribed within 24 hours, and any uncertainties were clarified with participants. No repeat interviews were conducted. Interview transcripts and study findings were not returned to participants for corrections or feedback.

**Table 3:** Overview of questions used in the interview guide.

Role	Interview questions
**Patients**	①Can you describe your first reaction when you experience loss of appetite or inability to eat? What measures have been taken?
	②Can you share your emotional experiences and changes in actions during disease development process that may require treatment with artificial nutrition and hydration?
	③What factors do you think affect your decision-making of artificial nutrition and hydration?
	④What impact do you think your current decisions regarding artificial nutrition and fluid replacement have had on you? Do you feel regretful?
**Family caregivers**	①Can you describe your first reaction when the patient in your care has a loss of appetite or inability to eat? What measures have been taken?
	②Can you share your emotional experience and changes in actions during the process when the patient in your care may require artificial nutrition and hydration?
	③What factors do you think affect your decision-making of artificial nutrition and hydration for the patient in your care?
	④What impact do you think your current decisions regarding artificial nutrition and fluid replacement have had on you or the patient in your care? Do you feel regretful?

### Data analysis

Data collection and analysis were conducted concurrently. Interview data were managed using NVivo 12.0 software and analyzed following Charmaz’s constructivist grounded theory methodology, which involves initial coding, focused coding, and theoretical coding. Analysis continued until theoretical saturation was reached. In accordance with grounded theory principles, all relevant data were considered.^25^ Notably, no prior relationship existed between the researcher and the participants before data collection. To enhance analytical rigor and minimize bias, two strategies were employed: constant comparison and questioning the data. After each interview, previously established codes were reevaluated and adjusted to maintain consistency and stability across the dataset.

In the initial coding stage, textual data were reviewed iteratively without reference to a predefined theoretical framework. Meaningful units such as “balancing the pros and cons of gastric tube” and “lowering the quality of life” were extracted and systematically organized. Memos were written to ensure the retention of critical concepts. This process resulted in the generation of 221 initial codes.

In the focused coding stage, frequently occurring, significant, or particularly insightful initial codes were synthesized to form broader categories. Simultaneously, the properties of each category were identified. For example, within the category of individual factors, properties such as cognition, emotion, and individual values emerged. Theoretical sampling was conducted at this stage to deepen understanding of these properties. Ultimately, eight subcategories and two main categories were identified.

In the theoretical coding stage, the relationships among the categories were examined in conjunction with memos to identify a core category. Analysis revealed that regardless of the influencing factors or how decision-making trajectories evolved, the shared priority among patients and family caregivers was to ensure patient comfort at the end of life. Therefore, “To achieve comfort” was identified as the core category, guiding the development of the final theoretical framework.

**Fig. 1:**
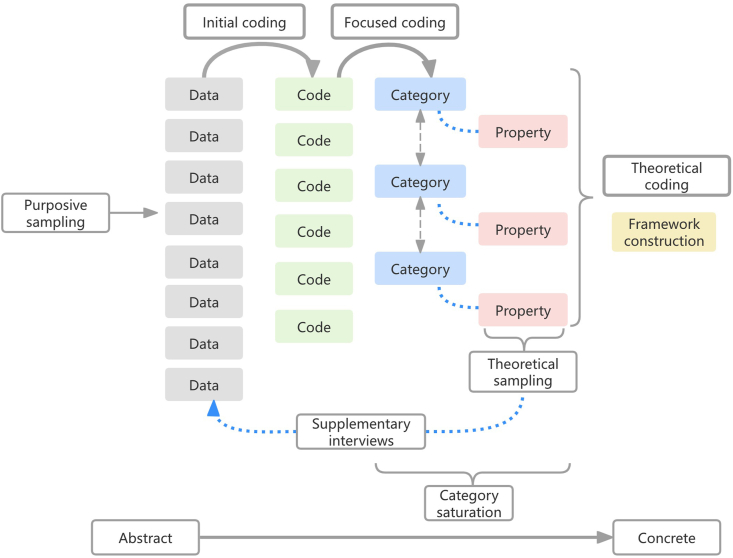
Data collection and analysis process.

To verify theoretical saturation, the research team conducted a thorough analysis of the final two interviews (M20 and M21) and compared their data with previously developed codes. The analysis showed that all newly generated data aligned with the existing 221 initial codes, and no new codes, categories or relational structures emerged. This confirmed that additional data no longer contributed to theory development, thereby establishing that theoretical saturation had been achieved.^24^ Fig. 1 illustrates the overall process of data collection and analysis. Table 4 shows an instance of concept formation.

**Table 4:** Instance of concept formation.

Text from the Interview	Code	Subcategory	Main category	Core category
My father has been losing weight continuously. Because he vomits whenever he eats, he has lost the desire to eat altogether. I Am very worried about this situation, and my father is also very anxious.	Experiencing helplessness	Symptom shock	The trajectory of decision-making of ANH during hospice care	To achieve comfort
I Would go out for a walk with my husband, relax at an agritainment resort, and eat organic food to improve his condition.	Changing the patient's living environment			
My main concern is whether the percutaneous endoscopic gastrostomy (PEG) procedure will be effective (pause, sigh). He is already so frail now, what if his body cannot withstand the surgery? That would mean more suffering and wasted money. But if it helps improve his nutrition, it would be worth it. Whether or not to proceed with PEG feels like a gamble.	Balancing the pros and cons of PEG	Risk trade-off		
After inserting the nasogastric tube, I had to restrain my father’s hands because he has Alzheimer’s. Sometimes, when he was confused, he would try to pull it out. We felt it was too cruel and uncomfortable for him. But without the tube, his lung infection could have been much worse.	Balancing the pros and cons of nasogastric tube			
All I could think was “I want to live”.	Better a living dog than a dead lion	Individual factors	Factors influencing decision-making of ANH in hospice care	
Although the tube maintained Grandma’s nutrition, she found it uncomfortable. So, we removed it.	Decreased quality of life			
All of my mother’s treatment costs were covered by the city’s health insurance, so it was manageable.	Social support system	Social factors		
My mum is very open-minded; she believes that death is Christ’s plan and that she’s on her way to heaven. She doesn’t want any intubation procedures. In fact, this morning, she even said she was done with treatment and just wanted to go home.	Sociocultural and religious beliefs.			

ANH, artificial nutrition and hydration.

### Rigor and trustworthiness

This study used credibility, reliability, confirmability, and transferability to ensure rigor.^26^ Credibility was established by strictly following Charmaz’s grounded theory methodology and the Criteria for Reporting Qualitative Research (COREQ)^27^ (Supplementary material). Besides, a detailed manual was developed to guide the interview process. To improve reliability, the first author (Li Y) was responsible for data collection and undertook systematic training in semi-structured interviewing techniques. Prior to each interview, the researcher reviewed and refined the interview guide as necessary. Additionally, to minimize researcher bias, she refrained from providing direct nursing care to patients. To ensure confirmability, data coding was performed by two researchers, and coding decisions were discussed in regular team meetings involving three nursing graduate students, one hospice clinical nurse, one nursing administrator, and one nutrition specialist. Any discrepancies were resolved through consensus. Finally, to achieve transferability, the study provides detailed descriptions of participant selection, data collection and analysis procedures.

### Ethical considerations

The study was conducted in accordance with the principles stated in the Declaration of Helsinki and was approved by the Clinical Research Ethics Committees of several institutions: The Affiliated Cancer Hospital of Nanjing Medical University (IRB No. KY-2024-086). Air Force Hospital of Eastern Theater and the Affiliated BenQ Hospital of Nanjing Medical University approved the study, based on the results of an ethical review by the Affiliated Cancer Hospital of Nanjing Medical University. Prior to interviews, participants were fully informed about the study’s purpose, process, and significance. They had the right to withdraw at any time, and their informed consent was obtained and recorded. To ensure confidentiality, all collected data were de-identified.

## Results

**Fig. 2:**
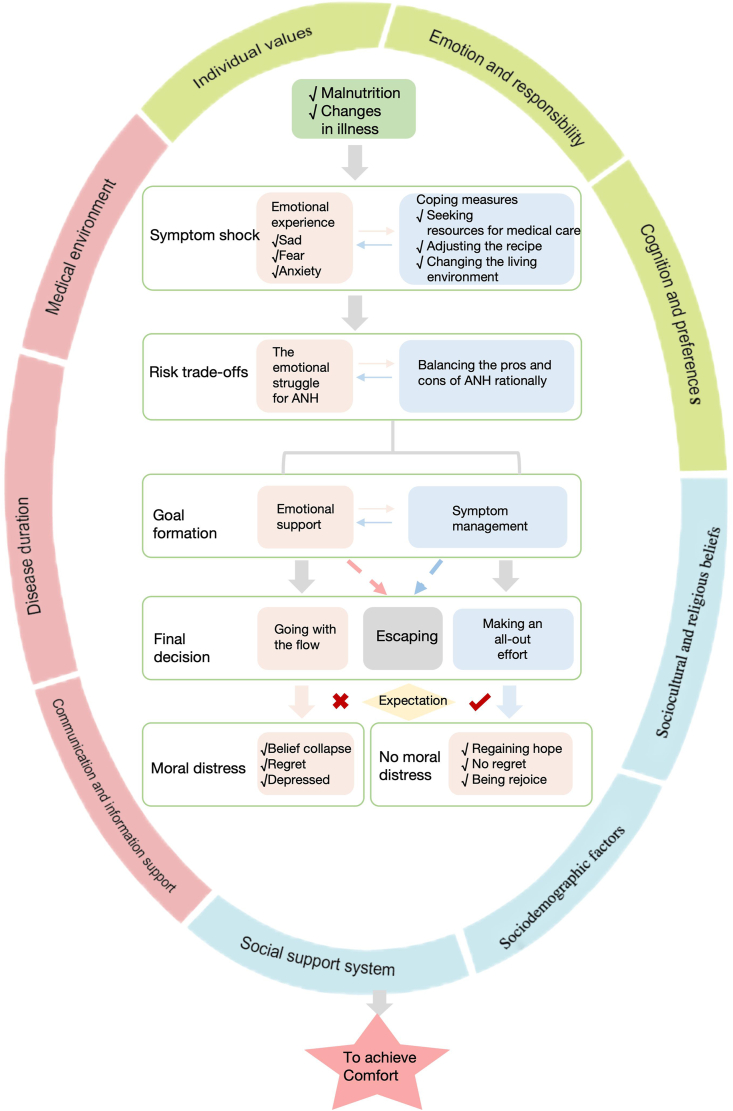
The decision-making process of ANH for hospice patients and family caregivers. ANH, artificial nutrition and hydration.

Using grounded theory methodology, this study identified one core category—“To achieve Comfort”—and two main categories: (1) The trajectory of decision-making of ANH in hospice care, and (2) Factors influencing decision-making of ANH in hospice care. Based on these findings, a conceptual framework was developed, titled “The decision-making process of ANH for hospice patients and family caregivers” (Fig. 2). The framework illustrates that the decision-making trajectory is a complex, evolving, and dynamic process shaped by multiple factors, with the ultimate goal being the comfort of the patient. The core category serves not only as the driving force behind the decision-making process but also influences how individuals weigh and respond to various factors at different stages. Within the main category “The trajectory of decision-making of ANH in hospice care”, five subcategories were identified: (1) Symptom shock; (2) Risk trade-offs; (3) Goal formation; (4) Final decision; and (5) Moral distress. These subcategories represent distinct phases in the decision-making process, with the salience of influencing factors varying across stages. Within the second main category—“Factors influencing decision-making of ANH in hospice care”—three subcategories emerged: (1) Individual factors; (2) Medical factors; and (3) Social factors. These factors interact to shape the trajectory of decision-making, contributing to transitions between different stages of the process.

### The trajectory of decision-making of ANH in hospice care

This decision-making trajectory began with the detection of malnutrition, and progressed through five sequential stages. In the early stages, emotional experiences significantly influenced rational behavior, which, in turn, reshaped emotional responses and guided the direction of decision-making in the subsequent stages, eventually leading to the formation of distinct goals. In addition, the psychological response experienced during the stage of “moral distress” further influenced the decision-maker’s future tendencies, creating a cyclical effect in the decision-making process.

#### Symptom shock

The decision-making process of ANH often began when a patient was diagnosed with malnutrition or experienced a sudden deterioration in their condition. This was typically accompanied by symptoms such as nausea and vomiting, dysphagia, or severe weight loss. When patients and their family caregivers first encountered these symptoms, they often experienced intense emotions such as sadness, anxiety, and helplessness.

“My father has been losing weight continuously. Because he vomits whenever he eats, he has lost the desire to eat altogether. I am very worried about this situation, and my father is also very anxious.” (M7)

These emotional responses prompted patients and family caregivers to adopt short-term coping strategies, such as seeking medical resources or changing the living environment, in an attempt to slow disease progression and mitigate physical decline.

“Intestinal adhesions made his stomach swell up like a drum and wouldn’t subside. The next day, I called my son and transferred him to a more advanced hospital.” (M1)

“I would go out for a walk with my husband, relax at an agritainment resort, and eat organic food to improve his condition.” (M19)

These actions served both as responses to emotional distress and as a foundation for subsequent decision-making.

#### Risk trade-offs

When short-term coping strategies failed to improve the patient’s condition, patients and family caregivers were compelled to confront the critical decision of whether to initiate ANH. At this stage, they often experienced emotional turmoil, fearing that their choices might not align with familial or personal expectations.

“If we withdraw ANH, we feared that our parents would think that we weren’t doing enough to treat our dad.” (M7)

“I worry that if things don’t turn out the way I hoped, I’ll regret this decision forever.” (M3)

Such emotional struggles led to more rational deliberations. A central concern emerged: whether ANH would genuinely improve the patient’s quality of life or merely prolong suffering.

“After inserting the nasogastric tube, I had to restrain my father’s hands because he has Alzheimer’s. Sometimes, when he was confused, he would try to pull it out. We felt it was too cruel and uncomfortable for him. But without the tube, his lung infection could have been much worse.” (M8)

As the rational analysis deepened, decision-makers gradually developed clearer goals, transitioning to the next stage.

#### Goal formation

Following emotional and rational considerations, decision-makers began to articulate their goals. These goals generally fell into two categories: some family caregivers prioritized the patient’s emotional well-being and aimed to maintain their mood and mental state at the end of life.

“Grandma’s wishes come first. Our priority is to keep her in good spirits; otherwise, what’s the point if she is unhappy every day?” (M11)

Others focused on practical problem-solving, especially with regard to meeting nutritional needs.

“Our main concern now is how to manage the nutritional issue or some other practical problems.” (M13)

Goal formation signaled a transition into a more action-oriented phase of decision-making.

#### Final decision

At this stage, decision-makers translated their goals into concrete behaviors, which typically fell into one of three patterns:

(1) Making an all-out effort: Some caregivers emphasized persistence and actively sought all possible means to extend the patient’s life.

“Even if it doesn’t make much sense anymore, we can’t abandon Grandpa and let him die at home while we do nothing.” (M12)

(2) Going with the flow: Family caregivers realized that preserving dignity, alleviating pain, and meeting emotional needs may be more important than simply prolonging life. They chose to minimize medical intervention.

“If he truly can’t eat anymore, I won’t force him. My approach now is simple, if he can eat, he eats. If he can’t, we’ll rely on intravenous infusion of nutrient solutions.” (M6)

Others shifted their focus from nutrition-related care to enhancing the patient’s comfort or quality of life.

“Usually her daughter would buy protein powder or some other nutritious food. She was also worried that the bright lights in the hospital room were disturbing her mom’s sleep, so she got her an eye mask to keep her comfortable.” (M11)

(3) Avoidance: Decision-makers struggled with internal conflicts, unable to decide whether to continue or withdraw ANH. As a result, they chose to defer the decision or follow the advice of others without actively engaging in the choice.

“I know that without eating, survival is impossible, but sometimes I don’t. I will just listen to my daughter.” (M9)

However, reaching a decision did not mark the end of the process. Patients or family caregivers often entered a phase of psychological adaptation.

#### Moral distress

The outcomes of decision-making of ANH not only affected patients' physical conditions but also shaped caregivers' moral reflections and psychological responses. When decisions aligned with their moral expectations, family caregivers often experienced peace and acceptance.

“At night, I remind myself that even though my mother now relies entirely on intravenous nutrition, her nutritional indicators seem stable. That gives me comfort.” (M14)

Conversely, when outcomes were less favorable, family caregivers might experience regret, self-blame, or emotional distress.

“My sister was always anxious and confused. She thought that if we had focused on my father’s swallowing function earlier, his life might have been extended. She regrets missing that opportunity.” (M2)

These psychological responses at this stage may influence future decision-making tendencies.

### Factors influencing decision-making of ANH in hospice care

This main category highlights that decision-making of ANH in hospice care is influenced by a combination of individual, medical, and social factors. These factors interact to influence both rational judgment and emotional experiences throughout the process, ultimately determining the final decision.

#### Individual factors

This study found that cognition and preferences, emotion and responsibility, and individual values are key individual-level factors influencing the decision-making of ANH. These elements reflect the personal wishes and psychological states of patients and their families.

(1) Cognition and preferences

At the stage of “risk trade-offs”, differing cognition and preferences often determine a family caregiver’s willingness to proceed with ANH. Some family caregivers viewed ANH as “force-feeding” and were more avoid or withdraw from it.

“If it reaches the point where she can no longer eat or drink, I wouldn’t consider inserting a gastric tube, It’s too painful. It’s better to let nature take its course.” (M11)

Conversely, those who perceived ANH as part of life-prolonging primary care or dietary support tended to advocate for its use.

“At the very least, this procedure (PEG) can sustain life, reduce suffering, and prolong survival.” (M4)

“At first, we all thought this gastric tube was my ‘lifeline’.” (M18)

Some patients strongly preferred “oral intake only” and were reluctant to rely on tube feeding to prolong their lives.

“I would never agree to have a gastric tube. I need to eat with my own mouth to maintain nutrition. If my illness reaches that stage, then let me go. I don’t want to endure such suffering.” (M20)

(2) Emotion and responsibility

Emotions were present throughout the process and were closely tied to a sense of moral duty. During the stage of “symptom shock”, family caregivers often felt anxious about the patient’s inability to eat and responded with an urge to take action, often turning to ANH.

“Seeing her so frail, as a family caregiver, I just want her to eat something. But since she can’t eat on her own, we have no choice but to rely on nutritional support.” (M14)

At the stage of “moral distress”, when ANH led to visible discomfort, caregivers experienced guilt and regret.

“I can’t say whether his condition improved or if he just became more uncomfortable after the gastric tube was inserted. But now, this gastric tube makes it difficult for him to speak clearly. When relatives visit, but can’t communicate, he just sits there, breathing through his mouth open. He looks so uncomfortable (sigh)!” (M15)

(3) Individual values

Individual values influenced how individuals balanced life extension and quality of life. Although they realized that ANH may diminish their quality of life, some terminally ill patients and caregivers adhered to the concept of “Better a living dog than a dead lion”. They still hoped to prolong their lives through this way.

“All I could think was ‘I want to live’.” (M21)

Others prioritized comfort and dignity over prolonged life, opting to forgo ANH.

“Although the tube maintained Grandma’s nutrition, she found it uncomfortable. So, we removed it.” (M11)

In some cases, patients chose ANH not for their own benefit, but to reduce the burden on their families.

“I feel nauseous every time I eat, but I don’t want to trouble my son to cook and bring me meals every day. He’s already very busy with work and taking care of my grandson, who’s preparing for college entrance exams. So, I eat less and rely on nutrient solutions.” (M10)

#### Medical factors

Medical factors such as communication, information support, medical environment, and disease duration provided practical foundations for decision-making and influenced rational judgment. These were especially salient during the stages of “risk trade-offs”, “final decision”, and “moral distress”.

(1) Communication and information support

Family caregivers often relied on HCPs for guidance, but some HCPs failed to initiate discussions about decision-making of ANH, leaving caregivers uncertain.

“We assumed the doctor would take the initiative in addressing my dad’s nutritional issues. I wouldn’t have taken the initiative to talk to the doctor first. Besides, I don’t even know how to communicate with doctors.” (M8)

When HCPs offered only binary choices without explaining the specific effects and long-term impacts of ANH, decisions were sometimes made from a position of ignorance.

“The doctor just asked if we wanted to insert a stomach tube but didn’t explain anything. I’m a farmer who doesn’t know much about these things, so I just decided against it.” (M5)

(2) Medical environment

Compared to the out-of-hospital settings, hospitals provided more resources and professional support, increasing the likelihood of choosing ANH during the stage of “final decision”.

“The hospice unit is well equipped with medical facilities. If my father needed nutritional tubes, oxygen, resuscitation machines, the nursing home wouldn’t have been able to provide them.” (M12)

However, conflicting recommendations between institutions sometimes complicated decision-making.

“Some doctors recommend PEG, while others say it was unnecessary. I don’t know what to decide.” (M16)

(3) Disease duration

As illness progressed, family caregivers' perspectives evolved from prolonging life to accepting natural death, enhancing their psychological resilience during the stage of “moral distress”. In cases where patients lived beyond their expected lifespan, families were more likely to accept discontinuation of ANH.

“When he first had surgery, the doctor estimated a five-year survival period. But now, he has lived for more than 15 years! Haha, I suppose my husband has already earned extra time, right? So, I am mentally prepared for him to give up nasal feeding.” (M15)

This illustrates that decision-making of ANH is not a single event, but a dynamic process continuously adjusted alongside disease progression.

#### Social factors

As external regulatory mechanisms, social factors not only influence the economic feasibility and moral acceptability of decisions but also shape how decision-makers respond throughout the process. These influences are primarily reflected in the following aspects.

(1) Social support system

Medical insurance and social welfare programs, as key components of the social support system, affect the affordability of ANH. During the stages of “risk trade-offs” and “final decision”, high costs may present a significant barrier for patients and their families.

“He receives most of his nutritional support through nutritional tube. The albumin is very expensive, and have to pay for it ourselves. We used to use it regularly, but now we’ve had to cut back.” (M15)

Conversely, a strong social support system can ease the financial and emotional burden of ANH.

“All of my mother’s treatment costs were covered by the city’s health insurance, so it was manageable.” (M14)

(2) Sociodemographic factors

During the stage of “goal formation”, age was found to influence family caregivers’ decision-making tendencies. Caregivers were more inclined to accept the withdrawal of ANH in elderly patients.

“He’s 85 years old, not a young man anymore. I’m okay with not giving him nasal feeding. But if he were in his 50s, we’d definitely do something about it.” (M15)

Additionally, families with higher incomes were more likely to pursue the “making an all-out effort” path at the final stage of decision-making, as their financial capacity allowed for continued use of costly nutritional support.

“We have been giving him intravenous nutrition solution. The nutrient solution was prepared by our daughter, which is expensive, but fortunately, she has a good job and can support us financially.” (M16)

(3) Sociocultural and religious beliefs.

Culture encompasses shared traditions, values, and religious beliefs within a society. In Chinese Confucian culture, discussions about death are often avoided. Consequently, decision-making of ANH are typically delayed until the late stage of illness, when patients are unconscious or unable to express their wishes. This may intensify the anxiety and uncertainty of family caregivers during the stage of “risk trade-offs”, and the resulting decisions may not align with the patient’s true preferences.

“We’ll think about ANH later, when the time comes.” (M6)

“We don’t usually talk about life and death; we reminisce about the past.” (M15)

Religious beliefs may help ease caregivers' emotional burden during the stage of “moral distress”. Certain belief systems embrace the concept of a “good death,” emphasizing comfort over life-prolongation through ANH. Patients influenced by such beliefs are more likely to adopt a “going with the flow” approach during the stage of “final decision”.

“My mum is very open-minded. She believes that death is Christ’s plan and that she’s on her way to heaven. She doesn’t want any intubation procedures. In fact, this morning, she even said she was done with treatment and just wanted to go home.” (M14)

## Discussion

### Main findings

This study developed a framework outlining the decision-making trajectory of ANH in hospice care from the perspectives of patients and family caregivers. The framework reveals that the decision-making of ANH is a complex, evolving, and dynamic process shaped by a combination of individual, medical and social factors. In pursuing the core category of achieving comfort, hospice patients and family caregivers adopt various coping strategies throughout the decision-making process. Compared to previous studies^28^^,^^29^ that primarily focused on the decision-making endpoint, this model deepens the understanding of the decision-making process by integrating both emotional and rational pathways, thus offering a novel perspective for theoretical advancement in this field.

Power et al.^30^ argued that health care decision-making often involves a dynamic interplay between rational cognition and emotional perception, with final decisions emerging from cognitive and emotional trade-offs in response to health threats. This study supports that view and further elucidates the shifts in emotion and cognition across different stages. Initially, patients or family caregivers experience the stage of “symptom shock”, confronting negative emotions associated with malnutrition and seeking more positive emotional states through rational action. They then move into the stage of “risk trade-offs”, where feelings of helplessness and fear prompt them to weigh the potential benefits and drawbacks of ANH. Subsequently, decision-makers form a goal—either emotionally driven by emotional support or rationally driven by symptom management. Finally, decision-makers reach the stages of “final decision” and “moral distress” during “goal formation”. Thus, HCPs should assess the decision-making trajectory dynamically to understand the emotional and cognitive states of hospice patients and their family caregivers. Tailored guidance and support should be provided to address individual needs and reduce potential deviations in the decision-making process caused by irrational thinking or emotional imbalance.

This study identified three distinct decision-making behaviors regarding ANH during the stage of “final decision”: “making an all-out effort”, “going with the flow”, and “escaping”. Importantly, when family caregivers choose “going with the flow”, it does not imply abandoning treatment. Rather, this approach reflects an understanding of the limitations of ANH, consistent with the findings of Hochwald et al.^17^ We further found that family caregivers expressed care through other comfort care that was more responsive to the patient’s needs, thus creating a psychological and emotional connection with the terminally ill patient, which in turn reduced their feelings of guilt or regret at the stage of “moral distress”. This suggests that HCPs should give positive feedback to family caregivers at the stage of “moral distress”, directing their attention from nutritional care to other forms of alternative care. Follow-up mechanisms should also be established to observe the decision-maker’s reaction to the outcome of the decision and intervene with psychological counseling if necessary. Future research could further focus on preventive measures for psychological trauma.

The framework for decision-making of ANH in hospice care demonstrates that a combination of individual, medical, and social factors significantly influences decision outcomes. Among these, individual factors serve as determinants, while medical and social factors play moderating roles. First, the cognition and preferences of decision-makers constitute the rational foundation for decision-making of ANH, reflecting both autonomous intentions and expectations regarding treatment outcomes.^31^^,^^32^ Values become a core factor at the stages of “goal formation” and “final decision”, where the individual’s prioritization of “quality of life” versus “length of life”, ultimately guides decision preferences. Notably, this study observed contrasting decisions by patients based on their consideration of family caregivers' quality of life. In previous research,^33^ some patients opted to forgo ANH to shorten the disease course and reduce caregiver burden. In contrast, this study found that some patients chose to maintain ANH to reduce the frequency with which caregivers had to prepare meals, thereby alleviating caregivers' workload. This highlights the need for HCPs to recognize individual definitions of quality of life and to provide tailored assessments when offering decision aids. Second, variations in communication patterns and managements across health care settings influence the quality of information provided to patients and their families, thereby affecting their decision-making experience.^34^^,^^35^ Inadequate or inconsistent information may heighten anxiety and confusion during the stages of “risk trade-offs” and “final decision”. A retrospective observational study in the United States found that families of long-term hospitalized patients tended to favor active treatment options.^36^ However, our study revealed that the long-term care experience led some caregivers to change their perceptions of end-of-life and become more accepting of the decision to withdraw ANH. The discrepancy between the two findings may be attributed to differences in disease duration and sample size of the subjects. Future research involving large-scale quantitative studies in mainland China could further explore how varying disease trajectories affect decision-making of ANH. Consistent with previous studies in Israel and Japan,^35^^,^^37^ this study confirmed that the social support system functions as a buffering factor that moderates financial stress associated with ANH, thereby facilitating more rational decision-making. In addition, it was found that elderly and religious patients and their families were more likely to accept the withdrawal of ANH, possibly due to their life experience and faith helping to reduce fear and resistance toward death. Consequently, HCPs should attend to the preferences, values, and psychological states of individual decision-makers, improve their understanding of ANH, and incorporate religious perspectives into personalized management plans. Furthermore, to mitigate disparities in resources and treatment across care settings, health care providers should explore alternatives such as telemedicine. At the policy level, strengthening financial support for hospice care could help ease the isolation and pressure patients and families face in making difficult decisions.

In Chinese culture, ANH is not merely a medical intervention but also serves as an emotional bond between terminally ill patients and their family caregivers.^38^ By providing ANH, family caregivers fulfill their perceived duty of “caring”, aiming not only to alleviate the patient’s discomfort but also to express love and emotional support. Even when HCPs do not recommend continued use of ANH from the perspective of patient comfort, caregivers may still choose to “make an all-out effort” during the final decision stage, driven by a strong sense of familial responsibility. In this study, sons and daughters from high-income families attempted to prolong their parents' lives and maintained ANH when financial resources allowed. However, a previous survey in South Korea^39^ indicated that high-income families were more likely to withdraw from ANH. This suggests that the relationship between family income and decision-making of ANH must be interpreted within the context of different sociocultural frameworks. In addition, attitudes toward death vary significantly across cultures. Western societies, with a more open discourse surrounding death, typically promote early communication about end-of-life decisions.^40^ In contrast, the death-related taboo rooted in Chinese Confucian culture often results in delayed decision-making. Furthermore, while Western individualism emphasizes the patient’s autonomy in making medical decisions, the collectivist orientation of Chinese culture entrusts the family with the primary decision-making role, often guided by familial moral standards.^41^ This may result in outcomes that do not necessarily reflect the patient’s true wishes. Therefore, HCPs should develop a comprehensive understanding of how Chinese cultural norms influence medical decision-making. It is essential to guide patients and family caregivers toward constructing advanced care plans early in the disease trajectory to prevent excessive medical intervention or delayed decisions. Simultaneously, the promotion of a patient-centered, shared decision-making model can help ensure patient involvement in care decisions when possible, thereby enhancing both the rationality and humanistic quality of hospice care.

### Implications for nursing practice and research

The framework developed in this study offers a theoretical basis for nursing practice in hospice care. Nurses should pay close attention to the emotional and cognitive states of patients and family caregivers at different decision-making stages, and provide phased, individualized support to help balance emotional responses with rational judgments, thereby reducing decisional distress. Shared decision-making should be promoted in clinical care, along with early initiation of advance care planning. For future research, the model should be further validated across different disease types and care settings, and used to guide the development of nursing interventions that enhance the effectiveness and precision of decision support.

### Limitations

The study has limitations that should be considered when interpreting its findings. First, all patient participants were diagnosed with gastrointestinal malignancies. The decision-making situations of this group, who more frequently face eating difficulties and nutritional intervention choices during the hospice phase, are representative and valuable for theoretical analysis. Constructing a preliminary theoretical framework based on this group is reasonable, which helps to depict the complete decision-making process. However, this also limits the applicability of the framework to other conditions, such as organ failure and neurodegenerative disorders. Therefore, the currently proposed framework for decision-making of ANH should be considered as a staged exploration model. Nevertheless, the inclusion of family caregivers whose care recipients had non-gastrointestinal diagnoses provides some complementary perspectives and enhances the diversity of decision-making contexts captured in the study. Second, the study’s sample size is a relatively small sample. However, we follow the theoretical saturation principle of grounded theory and believe that the sample size is sufficient to meet the study objectives. Third, the study data was collected at Grade IIIA hospitals in China, which may limit the generalizability of the proposed theoretical framework, especially as rural or primary care organizations may differ in terms of access to resources and decision-making pathways. However, in mainland China, Grade IIIA hospitals are the main institutions that carry out hospice care, with more systematic stages of illnesses and care concepts, and thus can provide basic experience for constructing theoretical models. Furthermore, the range of basic information of the participants in this study, such as literacy level, disease type, nutritional status and the number of children the patient had is considered an advantage in the study because these participants can better represent the views of this group. Therefore, we believe the study still provides valuable insights into the decision-making of ANH of hospice patients and their family caregivers. Future studies should aim to expand the sample size and scope to include a more diverse group of disease types and medical backgrounds. This will provide insight into the similarities and differences in decision-making of ANH under different medical resources, care concepts, and cultural environments, in order to further validate and expand the framework constructed in this study.

## Conclusions

This study employed grounded theory to construct a model depicting the decision-making trajectory of ANH in hospice care, providing a comprehensive, dynamic, and multidimensional framework for understanding the behavioral and emotional changes experienced by hospice patients and family caregivers in China. The framework highlights the influence of individual, medical, and social factors on decision-making of ANH in hospice care, emphasizing the complex interplay between emotional and rational considerations. It provides a scientific foundation for the development of decision-support programs in hospice care. Future research should further explore the interactions among individual, medical, and social factors, and promote the establishment of a “personal-hospital-community” tripartite care model to enhance the decision-making experience and improve the quality of hospice care.

## CRediT author statement

**Yunrong Li:** Conceptualization, Methodology, data collection, transcription and summarization, Writing – Original draft. **Yuan Ji:** Conceptualization, data collection, transcription and summarization, Writing – Review and editing. **Tiantian Wang and Bo Yang:** Conceptualization, Methodology, data collection. **Fulin Gao and Liuliu Zhang:** Conceptualization, Writing – Review and editing. **Guoren Zhou and Yun Zhao:** Conceptualization, Methodology, Funding acquisition, Writing – Review and editing. All authors have read and approved the final manuscript.

## Ethical statement

The study and was conducted in accordance with the 1964 Helsinki Declaration and its later amendments or comparable ethical standards and was approved by the Clinical Research Ethics Committees of several institutions: The Affiliated Cancer Hospital of Nanjing Medical University (IRB No. KY-2024-086). Air Force Hospital of Eastern Theater and the Affiliated BenQ Hospital of Nanjing Medical University approved the study, based on the results of an ethical review by the Affiliated Cancer Hospital of Nanjing Medical University. All participants provided written informed consent.

## Data availability statement

The data that support the findings of this study are available on request from the corresponding author. Data are not made public due to privacy and ethical restrictions.

## Declaration of generative AI and AI-assisted technologies in the writing process

No AI tools/services were used during the preparation of this work.

## Funding

This work was supported by the Elderly Health Research Program of 10.13039/100017962Jiangsu Commission of Health (Grant No. LKM2024058). The funders had no role in considering the study design or in the collection, analysis, interpretation of data, writing of the report, or decision to submit the article for publication.

## Declaration of competing interest

The authors declare no conflict of interest.
